# pH-Responsive Polymer Implants for the Protection of Native Mammals: Assessment of Material Properties and Poison Incorporation on Performance

**DOI:** 10.3390/polym15040878

**Published:** 2023-02-10

**Authors:** Kyle Brewer, Todd J. McWhorter, Katherine Moseby, John L. Read, David Peacock, Anton Blencowe

**Affiliations:** 1 Applied Chemistry and Translational Biomaterials (ACTB) Group, Centre for Pharmaceutical Innovation (CPI), UniSA Clinical and Health Sciences, University of South Australia, Adelaide, SA 5000, Australia; 2 School of Animal and Veterinary Sciences, University of Adelaide, Roseworthy, SA 5371, Australia; 3 Ecological Horizons Pty. Ltd., P.O. Box 207, Kimba, SA 5641, Australia; 4 School of Biological, Earth and Environmental Sciences, University of New South Wales, Kensington, Sydney, NSW 2033, Australia; 5 School of Biological Sciences, University of Adelaide, Adelaide, SA 5000, Australia; 6 Davies Livestock Research Centre, School of Animal and Veterinary Sciences, University of Adelaide, Roseworthy, SA 5371, Australia

**Keywords:** fluidised-bed spray coating, reverse-enteric, pH-responsive, sodium fluoroacetate, subcutaneous implant, polymer, toxic

## Abstract

Efforts to mitigate the effects of feral cats through the management of remnant or reintroduced populations of threatened species, are often unsuccessful due to predation by control-averse feral cats, or ‘problem individuals’. In order to target these animals, we have developed the Population Protecting Implant (PPI). PPIs are designed to be implanted subcutaneously in a native animal. If the animal is preyed upon, and the implant ingested by a feral cat, release of a toxic payload is triggered in the acidic stomach environment and the problem individual is eliminated. We introduce the first toxic implant incorporating the poison sodium fluoroacetate. Manufactured via fluidised-bed spray coating, toxic implants exhibited uniform reverse enteric coatings and low intra-batch variation. Toxic implants were found to exhibit favourable stability at subcutaneous pH in vitro, and rapidly release their toxic payload in vitro at gastric pH. However, limited stability was demonstrated in rats in vivo (~39–230 d), due to the use of a filament scaffold to enable coating and was likely exacerbated by metachromatic interactions caused by 1080. This work highlights that future development of the PPIs should primarily focus on removal of the filament scaffold, to afford implants with increased in vivo stability.

## 1. Introduction

Globally, invasive mammalian predators are likely the greatest threat to biodiversity. The majority (58%) of modern bird, mammal, and reptile extinctions have been linked to predation by only 30 invasive mammalian predators [1]. Feral cats (*Felis catus*) are one of the most prevalent and damaging of these invasive species. Almost half (63 or 44%) of the 142 species extirpated by invasive mammal species, were lost at least partially due to feral cats [1]. A further 430 species are threatened by feral cats [1], and invariably, their survival is dependent on the effective management of feral cat populations. Whilst the costs of biodiversity losses are incalculable, resource losses caused by the activity of feral cats and the costs associated with their management, have resulted in a considerable global economic burden. For example, the United States and Australia have, respectively, incurred approximately $46 and $19 billion (USD) in resource losses and management costs in the past 60 years [2,3]. Ongoing research into the effects of invasive mammalian predators such as the feral cat, their effective control, and the conservation of species threatened by their activity, is crucial to ensure the preservation of global biodiversity.

This research is most needed in Australia, where feral cats have had a staggeringly disproportionate effect on endemic mammal species, as they are estimated to kill ~456 million native mammals annually [4]. Terrestrial mammals within the critical weight range (0.035–5.50 kg) are most susceptible to predation, due to their ‘meal-size’ and naivety to introduced predators [5,6,7,8].

Current control methods are not universally applicable, due to factors such as landscape suitability (e.g., for exclosure fencing) or proximity to urban environments (e.g., for poison baiting) precluding their use, or crucially, due to the aversion of feral cats to existing control methods [9,10]. As a result, it is rarely possible to maintain a completely predator-free landscape, and ‘beyond-the-fence’ conservation is frequently impacted by the presence of control-averse problem-individuals. The survival of a reintroduced population is dependent on the eradication of feral cats from the reintroduction area, and the ongoing control of incursive individuals from outside the reintroduction area. Similarly, the management of feral cats is key to the survival of remnant populations of threatened mammals, where a single feral cat can decimate the remaining population. Problem individuals have disproportionate impacts on reintroduced and remnant populations [11,12,13], as the presence of a single problem individual can be enough to lead to ‘catastrophic predation’ [13]—where a population is completely depredated, causing the failure of the reintroduction program or loss of the remnant population [13,14,15,16,17,18].

Thus, there is a need to protect threatened mammal populations from problem individual predators, through the development of innovative and targeted control methods. One such method is the use of population protecting implants (PPIs) [19,20]. PPIs are subcutaneous implants with a pH-responsive coating and a poison core. These are small, poison implants designed for implantation into native mammals during reintroduction or monitoring programs, as a means of targeting problem individuals [20]. If a feral cat preys upon and consumes a ‘toxic Trojan’ [19] native mammal, it will also ingest the implant due to their specific feeding habits and the diminutive size of the implant [21]. The outer coating of the PPI remains intact and inert under the skin but breaks down in the acidic gastric environment of the feral cat’s stomach. This causes the rapid release of the encapsulated poison, and the death of the feral cat. By enabling the targeted removal of problem individual feral cats, the remaining native mammals in the reintroduced or remnant population are protected from catastrophic predation, and the likelihood of the program’s success is increased. Ultimately, PPIs are intended to equip predator-naïve native mammals with an artificial, population-level, anti-predator defence system.

Recently, we reported the prototypical development of the PPI [20], including the use of a pH-responsive, reverse enteric coating [22], and a non-toxic ‘core’, which will rapidly dissolve in the gastric environment of the feral cat (pH 1.6) [23,24]. The implants were manufactured via a batch-type fluidised-bed spray coating method. Beginning with a substrate that enabled spray coating—a small piece of cylindrical ABS filament—a core coating was applied that contained a non-toxic (sodium acetate, NaOAc) formulation [20]. This was followed by a pH-responsive copolymer coating, that was soluble at pH ≤ 1.5, but insoluble at pH ≥ 2.0 (i.e., a reverse enteric coating), due to the protonation (at low pH) of the basic 2-vinylpyridine moieties in the copolymer structure. Our previous work demonstrated the proof-of-concept function of the PPI; non-toxic PPIs exhibited promising stability (12 months) and release (~1.5 h) properties in vitro, in respective simulated subcutaneous (pH 7.4) and gastric environments (pH 1.5) [20]. In addition, non-toxic PPIs demonstrated favourable stability in vivo (12 weeks) and were found to exhibit comparable biocompatibility to conventional radiofrequency identification (RFID) microchips. Given that poison implants with triggered release have not been previously reported, the PPI platform requires evaluation when a toxic core formulation is used, to determine whether the favourable stability and release properties of the implants are conserved in the presence of the poison. Optimal in vivo stability for the toxic PPIs would be a period equal to the life of the implanted mammal, to maximise the protection it affords.

In Australia, contemporary lethal control of feral cats via poisoning is primarily conducted using poison baits containing either sodium fluoroacetate (also known as 1080) or *para*-aminopropiophenone (PAPP) [25,26,27,28,29,30]. Native non-target species in Australia—particularly herbivores [31,32,33]—exhibit a high resistance to 1080, due to a co-evolutionary history with fluoroacetate-bearing vegetation [34,35]. 1080 also poses a reduced risk to unadapted native birds and reptiles due to their metabolic differences [32,33,36]. As a result, 1080 is highly effective against—and affords selectivity for—unadapted, invasive mammalian carnivores such as the feral cat (LD_50_ = 0.28 mg·kg^−1^) [37,38]. PAPP exhibits similar selectivity for mammalian carnivores [39,40]. However, a key design consideration in the development of the PPI was its manufacture to a size compatible with conventional RFID microchip syringe implanters.

RFID microchips are commonly used to identify and monitor mammals, which enables the PPIs to be incorporated with minimal additional training or changes to current processes. In addition, the diminutive size of an RFID microchip or similarly sized PPI, would be unlikely to affect the movement of an implanted animal. These considerations constrained the loading capacity of the PPIs, and only a 3 mg dose of poison (per implant) could be incorporated. Whilst 3 mg is a sufficiently lethal dose of 1080 for a feral cat [38], it is significantly lower than that required for PAPP (e.g., commercial baits contain 78 mg of PAPP) [30]. Therefore, for the use of PPIs in Australia, 1080 is the most pragmatic choice, due to its lethality and favourable selectivity. As a result, PPIs bearing a toxic, 1080 core formulation, and the previously reported reverse enteric coating [20], were manufactured. The in vitro and in vivo performance of the resultant 1080 PPIs was then evaluated. 

## 2. Experimental Section

### 2.1. Materials

All reagents were used as received unless otherwise specified. Analytical grade ethanol (100% undenatured), potassium chloride (KCl), hydrochloric acid (HCl, 36.5–38.0%), orthophosphoric acid (H_3_PO_4_), sodium acetate (NaOAc), toluene, and HPLC-grade methanol (MeOH; Honeywell) were purchased from Chem-Supply (Gillman, Australia). Pluronics F127 and poly(ethylene glycol) (PEG 10 kDa, *M_n_* = 10 kDa), methylene blue (≥82%), and tartrazine (80%) were purchased from Sigma-Aldrich (St. Louis, MO, USA). Sodium fluoroacetate (1080, 93%) was purchased from Animal Control Technologies Australia (ACTA, Somerton, Australia). Phosphate buffered saline (PBS) tablets were purchased from Sigma and used as instructed by the manufacturer. High-purity water (≥18.2 MΩ·cm) was produced by an Arium Pro Ultrapure Water System (Sartorius, Goettingen, Germany). pH 1.0 and 1.5 release solutions were prepared as outlined in the United States Pharmacopeia (USP29). Ethyl oleate (EO) and poly[(2-vinylpyridine)-*co*-(butyl methacrylate)-*co*-(isobornyl methacrylate)] (PVBI) were synthesised as previously reported [20]. Cylindrical acrylonitrile butadiene styrene (ABS) rods (0.55 mm diameter × 250 mm length) were purchased from BNA Model World (Moorabbin, Australia). Sterile microchip syringe implanters complete with standard microchips (2.12 mm diameter × 12 mm length) were purchased from Faread Technology Co. Ltd. (Jiaxing, Zhejiang) and used as received or loaded with a manufactured implant. 1.75 mm Polylite^TM^ poly(lactic acid) (PLA) 3D printing filament (Polymaker, Shanghai, China) was purchased from 3D Printing Solutions (Para Hills West, Australia). Mini microchips and syringe implanters (1.4 mm × 8.5 mm) were donated by Microchips Australia Pty Ltd. (Keysborough, Australia). Nitrogen and argon (≥99.999% purity) were purchased from BOC Ltd. (North Ryde, Australia).

### 2.2. Instrumentation

An Evolution 260 Bio spectrophotometer fitted with a single cell Peltier system (Thermo Scientific, Waltham, MA, USA) was used for ultraviolet-visible (UV-Vis) spectrophotometry. A benchtop Mini Coater/Drier 2 (Caleva Process Solutions Ltd., Dorset, UK) was used for fluidised-bed spray coating. Embedded implants were microtomed using a Microm HM 325 (Thermo Scientific, Waltham, MA, USA) fitted with a tungsten carbide knife. Implant cross-sections were imaged with an Olympus SZ61 stereomicroscope. A Gryphax Arktur (Jenoptik AG, Jena, Germany) camera module was operated with the Gryphax software (v2.2.0.1234) package and used to capture images of cross-sectioned implants during observation. ImageJ software (v1.53g) was used to analyse the images. Scanning electron microscopy (SEM) was performed on a Merlin Field Emission Scanning Electron Microscope (FE-SEM) (Zeiss, Oberkochen, Germany). SEM micrographs were contrast-adjusted in Adobe Photoshop (v22.4.1). Mechanical testing was conducted using an EZ-Test EZ-LX (Shimadzu Corporation, Kyoto, Japan) mechanical tester. High-performance liquid chromatography (HPLC) was conducted using a Shimadzu Prominence LC system (Shimadzu Corporation, Kyoto, Japan) fitted with a Shimadzu pump (LC-20AD), autosampler (SIL-20AHT) and photodiode array detector (SPD-M20A), and fitted with an Inertsustain Cyano column (3 µm, 4.6 × 250 mm) (GL Sciences, Tokyo, Japan). Computed tomography (CT) images were captured using a Toshiba Alexion Advance Edition 16 slice CT scanner and processed in Adobe Photoshop (v22.4.1). For further details, and a comprehensive description of the instruments, parameters and conditions refer to Brewer et al. [20].

### 2.3. Methods

#### 2.3.1. Preparation of Coating Formulations

The 1080 core formulation was prepared by dissolving PEG 10 kDa (15.0 g), 1080 (15.0 g) and methylene blue or tartrazine (400 mg) in 64/36% *v*/*v* EtOH/ultrapure water, under stirring. The temperature-controlled fluidisation air flow was set to 40 °C during coating.

The pH-responsive copolymer formulation (PVBI/EO_10_) was prepared by dissolving PVBI (9.00 g) and EO (1.00 g) in toluene (100 mL) under stirring to afford a 10% *w*/*v* solution. The fluidisation air flow was set to ambient temperature (23 ± 1 °C) during coating.

#### 2.3.2. Implant Manufacture

Manufacture of the 1080 core implants was conducted as previously reported [20], with the following exceptions. ABS filament scaffolds (0.63 ± 0.06 mm diameter and 7.39 ± 0.08 mm length, *n* = 1000), were coated with the 1080 core formulation. ABS scaffolds were coated in 45 min cycles, until ~6 mg of the core formulation had been deposited (i.e., equivalent to a 1080 dose of ~3 mg/implant). A curing step was employed following coating, where implants were dried in vacuo (16 h, 0.1 mbar, 23 ± 1 °C) and then stored in a desiccator prior to use. Core coated implants were coated with the PVBI/EO_10_ coating using the same approach, until a desired thickness was reached (i.e., 100, 200, and 300 µm). The copolymer-coated implants were dried in vacuo following each PVBI/EO_10_ coating cycle, and stored in a desiccator prior to use.

#### 2.3.3. In Vitro Stability Studies

In vitro stability studies were conducted in a 25% *w*/*w* Pluronics F127 hydrogel in PBS at pH 7.4, as previously described [20]. Implants (*n* = 5) were completely submerged in the hydrogel (3.5 mL) and monitored daily for their stability, with failure indicated by the presence of dye (methylene blue or tartrazine) in the hydrogel.

#### 2.3.4. In Vitro Release Studies

In vitro release experiments were performed as previously reported [20], with the following exceptions. Implant release in pH 1.0 and 1.5 solutions was monitored inline using UV-Vis spectrophotometry, via the presence of methylene blue (λ = 664 or 600 nm) or tartrazine (λ = 435 nm) in the release solution.

#### 2.3.5. In Vitro 1080 Diffusion Study

1080 core and PVBI/EO_10_-coated implants (100, 200, and 300 µm, *n* = 3) were submerged in PBS (pH 7.4, 3.5 mL) and stored at 37 °C. An aliquot (1 mL) of the PBS receiving solution was removed weekly for HPLC analysis, and at each sampling interval, the removed aliquot was replaced with fresh PBS solution (1 mL). Receiving solution aliquots were analysed via HPLC using an isocratic mobile phase consisting of 0.1% *v*/*v* orthophosphoric acid (H_3_PO_4_) in 1:1 *v*/*v* water:MeOH. Elution was conducted at a flow rate of 0.4 mL·min^−1^ over 18 min. All analyses were performed at ambient temperature (23 ± 1 °C), with an injection volume of 10 µL of the receiving solution sample. The presence of 1080 was determined via its absorbance (λ = 220 nm, retention time (R_t_) = 8.4 min) in the receiving solution. The amount of 1080 was quantified via a peak area comparison between the sample and a five-point calibration curve prepared from the analysis of standard solutions of 1080 (28–448 ppm) prepared in PBS (pH 7.4).

#### 2.3.6. In Vivo Stability Study

Animal work was conducted under approval S-2015-176 from the University of Adelaide Animal Ethics Committee. 1080 implants with a 300 µm PVBI/EO_10_ coating (*n* = 7) were sterilised using an ultraviolet light source (UVC, 30 min exposure). Sterile syringe implanters (2 mm) were loaded with a 1080 implant using aseptic techniques and vacuum sealed in storage bags until use. Male Sprague-Dawley rats (10–11 weeks of age, *n* = 7) were each subcutaneously implanted with a mini microchip for identification (superficial to the right scapula) and a single 1080 implant (superficial to the left scapula). Sterilised implants and microchips were administered under general anaesthesia via syringe implanters as previously described [20]. Anaesthesia was induced using a combination of interperitoneally administered medetomidine (0.1 mg·kg^−1^) and fentanyl (100–200 µg·kg^−1^), and maintained with isoflurane (1–2%, oxygen flow 300–600 mL·min^−1^). All rats were held on a warming pad during recovery. All 1080 implants and microchips were implanted at day 0, with rats checked visually daily and weighed weekly. Rats were scanned via CT at implantation, every 6 weeks thereafter, and at the conclusion of the experiment. Rats were monitored daily, and if exhibiting symptoms consistent with 1080 poisoning (e.g., ataxia, tremors, hunched posture, and hypothermia), were immediately euthanised. Rats were euthanised using CO_2_ and a post-mortem examination was conducted where implants were exposed and photographed prior to explantation then imaged thereafter via optical microscopy.

#### 2.3.7. 1080. Residue Testing

Rats #1 and #2 exhibited symptoms consistent with 1080 poisoning and were euthanised. Liver samples from rats #1 and #2 were analysed for 1080 residue by the Chemical Residue Laboratory at the Department of Agriculture and Fisheries (Queensland Government), using their in-house 1080 analysis method.

### 2.4. Statistical Analyses

All of the results are expressed as mean ± std. dev. Unpaired, two-tailed Student’s *t*-tests were used to compare the flexural strength and flexural modulus results for NaOAc and 1080 implants. *p*-values < 0.05 were considered significant. Minitab 17 (v17.1.0) was used for all statistical analyses. Exact *p*-values, t-values and degrees of freedom are summarised in the Supplementary Information Table S1.

## 3. Results and Discussion

### 3.1. Implant Manufacture

PPIs containing a 1080-loaded core and a pH-responsive copolymer coating were manufactured via sequential fluidised bed spray coating as previously reported for non-toxic PPIs with NaOAc-loaded cores [20]. Initially, a 1080 core formulation containing 1080 (instead of NaOAc), PEG (binder) and methylene blue (dye) (in a mass ratio of 1:1:0.026) was spray coated onto ABS filament scaffolds (*n* ≈ 1000). Of the resultant 1080-implant cores, ≈500 were then spray coated with a pH-responsive copolymer formulation containing PVBI and EO (10 wt%) (PVBI/EO_10_), as previously described [20]. The physical dimensions of the implants at various stages of manufacture were measured using digital callipers and point-to-point measurements of epoxy-embedded, microtomed implants (Table 1). The 1080 implants exhibited low intra-batch variability (≤8%) for all digital calliper measurements of diameter and length, consistent with that observed for the NaOAc implants [20]. Importantly, the mass of the 1080 core coating was found to exhibit low variability (8.0%), which demonstrated that an accurate and lethal dose of 1080 (3.12 ± 0.04 mg, >LD_99_ for feral cats) could be incorporated despite the large batch size of ≈1000 implant cores.

Calliper measurements of the thickness of the PVBI/EO_10_ coating showed little variability for all coating thicknesses (<10%) and were consistent between the middle (i.e., side midpoint) and ends. Compared to measurements made at the middle of the implants (≈2.4–5.6%), the coating thicknesses at the ends of the implants showed greater variability (≈6.0–9.9%). This difference likely resulted from a combination of the variability in length of the ABS filament scaffolds (1.1%) inherent to the manual cutting of the filament, and the difficulty of manually measuring the centre of each curved end. In comparison, the point-to-point measurements obtained from epoxy-embedded and microtomed implants were generally consistent with the calliper measurements, with a maximum percentage difference of ≈19%. 

**Figure 1:**
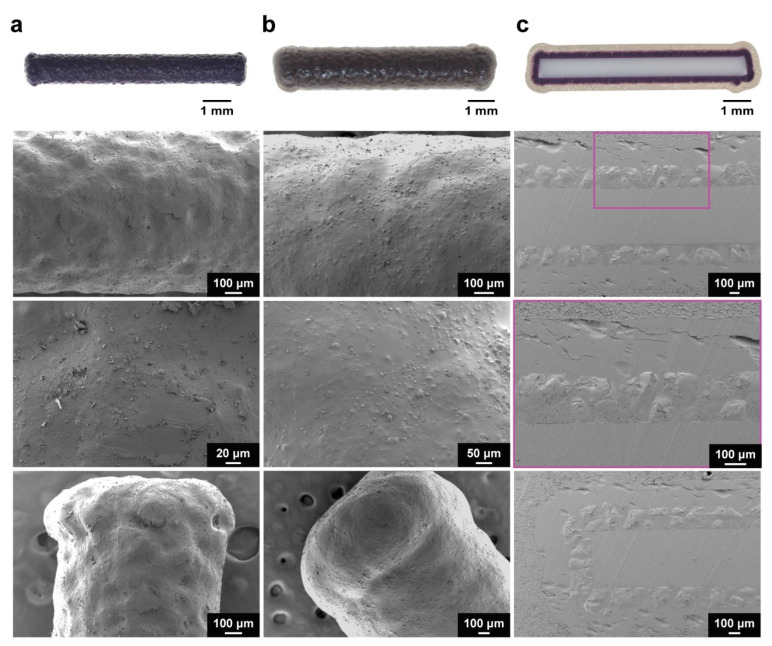
Representative digital images and scanning electron micrographs of (**a**) a 1080 core-coated implant, (**b**) a 1080 core and PVBO/EO_10_-coated (300 µm) implant, and (**c**) an epoxy-embedded and microtomed 1080 core and PVBI/EO_10_-coated (300 µm) implant; note the ‘flared’ ends of the core and polymer coated implants.

SEM analysis of the implants following coating with the 1080 core formulation revealed less pitting and an absence of cracking as compared to the NaOAc implants [20]. (Figure 1a and Supplementary Materials Figure S1). Though the exact mechanism is unclear, this effect was attributed to the complexation of PEG with free Na^+^. In solution, Na^+^ are known to cause a concentration-dependent reduction in solution viscosity, due to the complexation of Na^+^ with PEG chains [41]. In the presence of NaOAc, the rapid evaporation of ethanol would lead to the deposition of viscous, mostly aqueous droplets on the substrate surface, which could trap air bubbles with continued evaporation. However, facilitated by the greater dissociation—and, therefore, increased free Na^+^ concentration in solution—of 1080 (*cf.*, NaOAc), the reduction in solution viscosity likely prevented the trapping of air bubbles in the deposited droplets, thereby reducing pitting. In addition, light dusting was observed on the 1080 core-coated implants, which was believed to be platelet-like 1080 crystals deposited upon the surface. Despite this, the 1080 core-coated implants exhibited a qualitatively superior coating. 

SEM micrographs of the implants also revealed flared ends (Figure 1a) in contrast to the comparatively rounded NaOAc core-coated implant ends (Supplementary Materials Figure S1). This change in end morphology occurred during coating of the 1080 core formulation and was attributed to a build-up of electrostatic charge on the implant surface during coating. The triboelectric generation of electrostatic charge due to collisions between implants, and implants with the walls of the coating chamber, could have caused an accumulation of charge on the surface of the implants [42]. In contrast with commonly coated substrates, such as pharmaceutical tablets or capsules, which generally have rounded/smooth morphologies, the ABS filament used in this work was cylindrical and exhibited sharp edges at each end. It is likely that the morphology of the implants caused the localisation of charge at the ends of the implants, resulting in the electrostatic attraction of the coating formulation to the implant ends, and over several coating cycles, caused the disproportionate coating of the 1080 core formulation at the edges. It is likely that this effect was due to the incorporation of 1080, as it was not observed when coating was performed with the NaOAc core formulation and may be related to the differences in dissociation of the two compounds. Despite this, uniform, cohesive coatings were observed (Figure 1b), with only minor dusting occurring upon the implant surface. In addition, optical microscopy, and SEM analysis of epoxy-embedded and microtomed implants (Figure 1c) showed that the thickness and uniformity of the 1080 core coating were unaffected by the flaring. The PVBI/EO_10_-coated 1080 implants exhibited slightly wider ends due to the coating of PVBI/EO_10_ over the flared ends of the 1080 cores. 

### 3.2. Mechanical Testing

**Figure 2:**
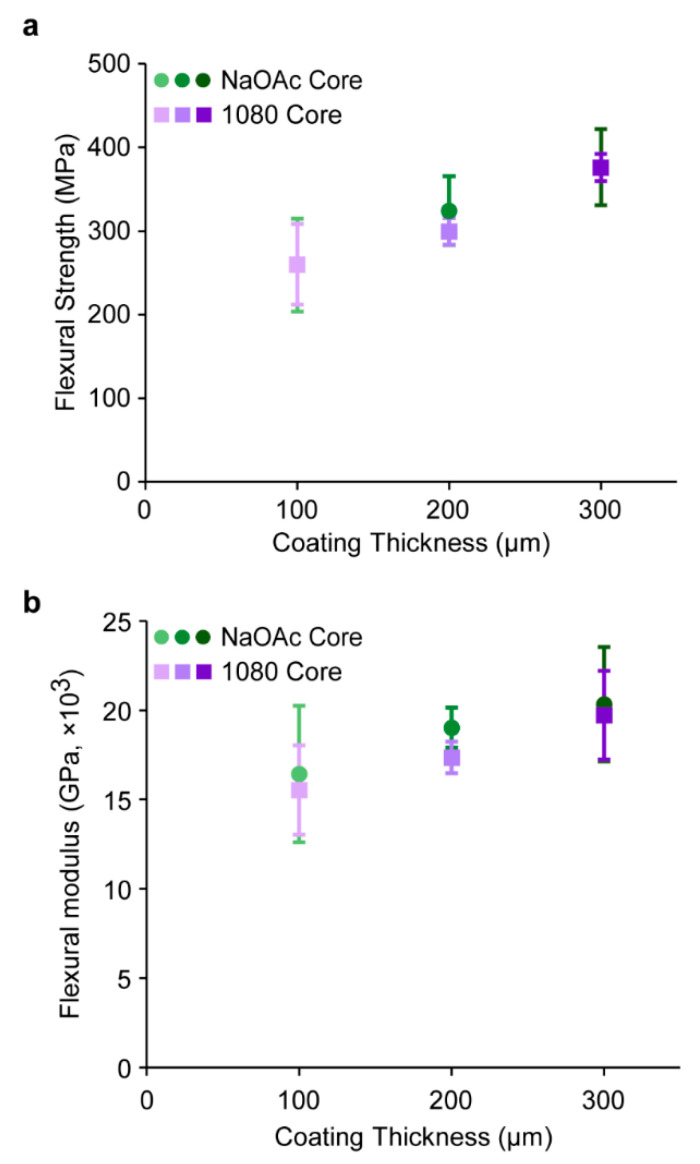
(**a**) Flexural strength (σ_F_) and (**b**) flexural modulus (E_f_) measurements of PVBI/EO_10_ -coated NaOAc and 1080 core implants, determined via three-point flexural testing (Supplementary Materials Figure S2). All tests were performed at 23 ± 1 °C, and all values are reported as the mean ± std. dev. (*n* = 5), *p* < 0.05 × 2-sample *t*-test of difference = 0 (vs ≠ 0) (Supplementary Materials Table S1).

The mechanical properties of the 1080 core implants were measured to investigate the effects of the incorporation of 1080 in the core formulation. Flexural testing was conducted on 1080 core implants, and the flexural strength (σ_F_) and modulus (E_f_) were determined as previously reported, using Equation S1 and Equation S2, respectively [20,43,44]. The flexural testing results of the 1080 core implants were compared to that of the NaOAc core implants [20]. Though a moderate increase in σ_F_ was observed as the PVBI/EO_10_ coating thickness increased from 100–300 µm, no significant difference was observed between the 1080 or NaOAc cores implants at each PVBI/EO_10_ coating thickness (Figure 2a). The similar σ_F_ of the 1080 and NaOAc core implants supported the assertions made in our previous work that the major contributor to implant σ_F_ was the PVBI/EO_10_ coating and its thickness, and not the composition of the core [20].

Similarly, no significant difference in E_f_ of the 1080 implants (*cf.*, to NaOAc core implants) was observed (Figure 2b), nor was an increase in E_f_ observed with respect to the PVBI/EO_10_ coating thickness, which suggested that the incorporation of 1080 afforded no change in the mechanical properties of the implant core. This was also consistent with our assertion that the core composition was the dominant contributor to the E_f_ of the implants. The similar E_f_ of the 1080 and NaOAc implants was likely due to the comparable bulk properties of both solid materials, resulting in similar mechanical properties once incorporated into the implant core. 

### 3.3. In Vitro Release

**Figure 3:**
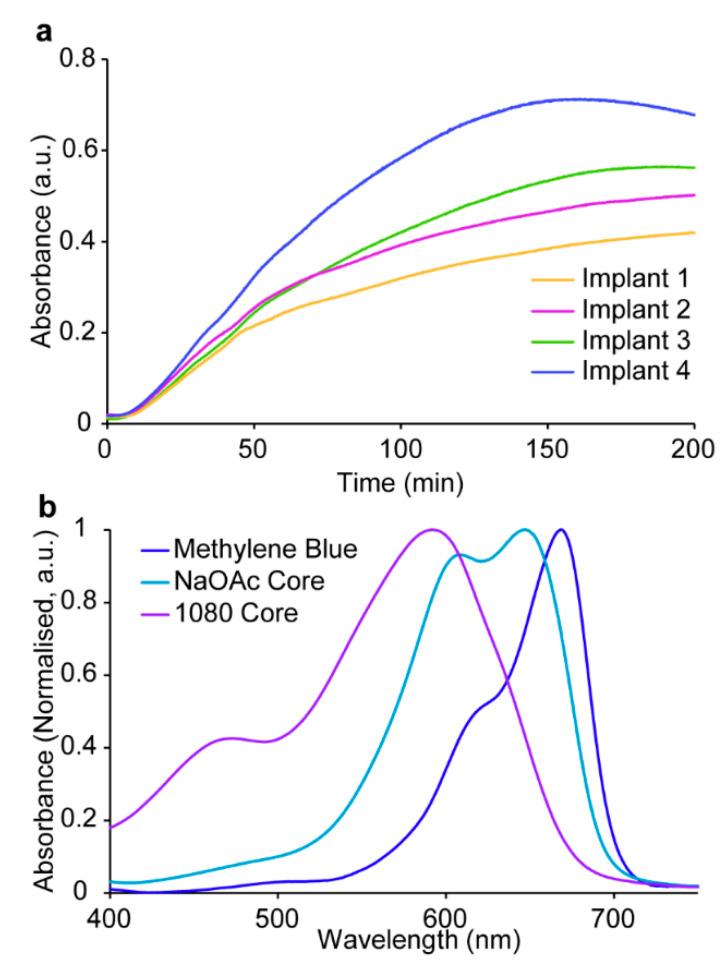
(**a**) Release profiles for implants containing a 1080 core and 300 µm PVBI/EO_10_ coating at pH 1.5, 37 °C (*n* = 4). (**b**) Comparison of normalised absorbance spectra of NaOAc core and 1080 core implants, and methylene blue solubilised in a pH 1.5 solution.

The dissolution characteristics of the 1080 implants under simulated gastric conditions were investigated in vitro at pH 1.0 and 1.5 by monitoring dye (methylene blue) release from the core via UV-Vis spectrophotometry [20]. However, preliminary testing revealed a marked change in the release profiles of tested implants, with a protracted and inconsistent release observed (Figure 3a). In addition, receiving solutions obtained following release appeared purple, in contrast to the blue receiving solutions obtained from NaOAc core implants. These observations suggested a change in the photophysical properties of methylene blue in the receiving solution, when solubilised from the NaOAc and 1080 core formulations at low pH. UV-Vis spectrophotometry revealed a blueshift in the absorbance maximum of methylene blue from 669 nm in a neat solution, to 647 nm in the NaOAc core solution, and 592 nm in the 1080 core solution (Figure 3b). Furthermore, there were also significant changes in other absorption bands of the spectra.

This phenomenon is known as metachromasy, a hypsochromic shift (also known as a blueshift) caused by the aggregation of charged dye monomers—in this case methylene blue cations—in solution [45,46]. The aggregation of dye molecules can be induced under a variety of conditions, including increased dye concentration, solution pH, dye adsorption to polyelectrolytes, addition of salts, and changes in the dielectric constant of the solvent [45,46,47,48,49,50,51]. In aqueous solutions, monomeric methylene blue cations exhibit an absorbance maximum at 665 nm (*n*–π*). In contrast, dimers and trimers have been reported to exhibit respective absorbance maxima of 610 and 572 nm due to the formation of H-type aggregates, and the resulting hypsochromic shift of the *n*–π* transition [51,52]. Recently, the formation of tetramers has also been proposed [53].

The observed metachromasy of methylene blue in release solutions from the NaOAc and 1080 core solutions was attributed to the presence of their respective anions in solution. When present in the core solution, NaOAc caused the formation of some dimers (608 nm) whereas 1080 almost exclusively caused the formation of trimers (592 nm). No significant difference in the absorbance maxima was observed for PVBI/EO_10_ coated implants analysed under the same conditions, suggesting that neither the presence of PVBI nor EO affected the aggregation of methylene blue, which provided further indication that NaOAc and 1080 caused the observed metachromasy. The significant aggregation of methylene blue in the presence of 1080 implied that there was a complex association between the two that is absent for NaOAc. The specific mechanism underlying the metachromasy observed in these experiments was outside of the scope of this work and further investigation was not undertaken. Instead, it was hypothesised that monitoring release of the 1080 core implants via the absorbance at 592 nm would ameliorate this issue. 

**Figure 4:**
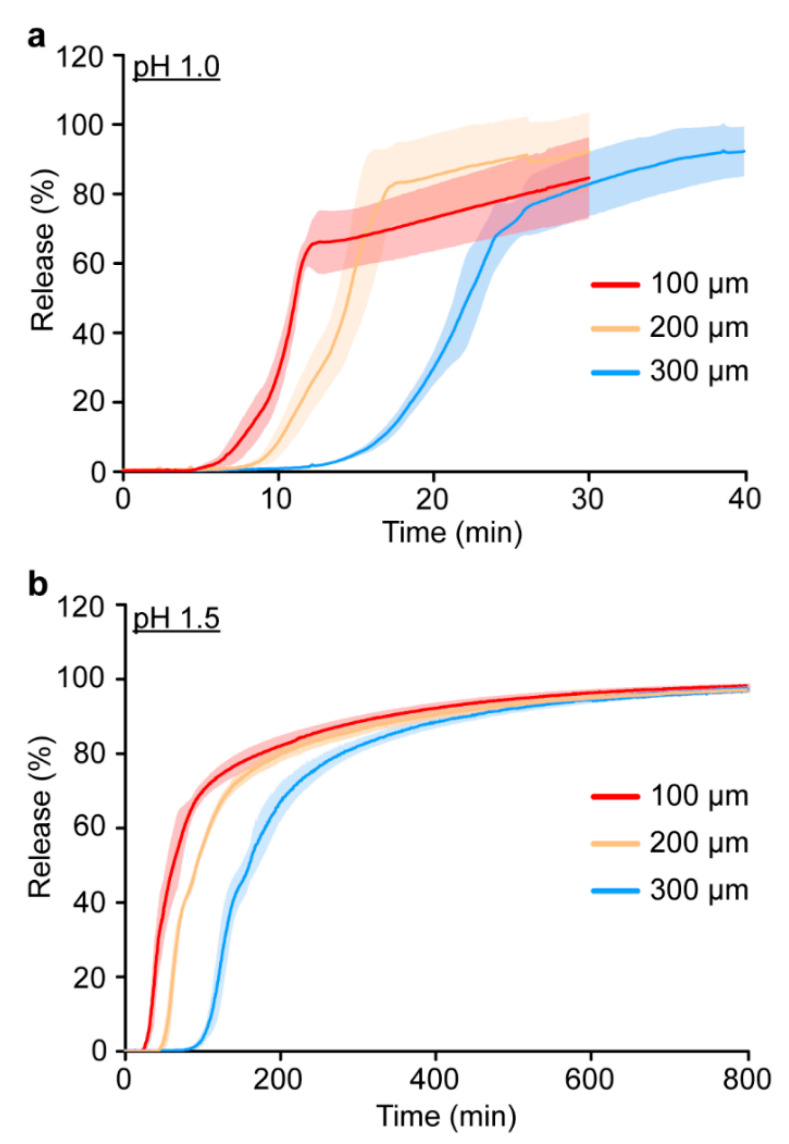
Release monitoring profiles of methylene blue from 1080 core and PVBI/EO_10_-coated implants (100, 200, or 300 µm) at 37 °C, in (**a**) pH 1.0 and (**b**) pH 1.5 release media (3.5 mL). Traces are reported as the mean (solid line) ± std. dev. (shaded area) (*n* = 5).

The release of methylene blue at pH 1.0 and 1.5 (Figure 4 and Table 2) displayed the same overall trends as previously reported for the NaOAc implants (Supplementary Materials Table S2) [20]. The initial and complete release times for the 1080 implants were proportional to both the PVBI/EO_10_ coating thickness, and pH of the receiving solution. Considering that the 1080 implants were manufactured with the same PVBI/EO_10_ coating (*cf.*, NaOAc implants), similar release characteristics were expected. However, whilst the initial release times (Table 2) were consistent with those of the NaOAc implants (Supplementary Materials Table S2) [20], complete release times for the 1080 implants at pH 1.0 and 1.5 were ~2 to 3 times greater. This was observed as a protracted release profile in both receiving solutions and for all coating thicknesses, and the measured absorbance appeared to gradually increase at later timepoints despite the implant being completely solubilised. 

The protracted release profiles were attributed to metachromasy and suggested that there was a temporal characteristic to the interactions that made monitoring more complex than first thought. Attempts to reproduce the effect by combining the core components (i.e., NaOAc or 1080, PEG 10 kDa, and methylene blue), and solubilising them under the same conditions (i.e., concentration, temperature, and pH) were unsuccessful. Interestingly, this suggests that an additional structural or temporal factor—perhaps introduced during the spray coating, or during solubilisation of the core coated implant—was playing a key role. It was hypothesised that the aggregation of methylene blue was caused by the ethanolic spray coating solution, as similar solvatochromic effects have been reported [54]. These aggregates could be released into the low pH solution without dissociating and potentially undergo further aggregation thereafter. The difficulty in reproducing the observed metachromasy appeared to support this postulation. 

The use of an alternative dye with similar solubility in the core formulation and no reported metachromasy, was hypothesised to mitigate these issues. Therefore, the 1080 core formulation was revised and methylene blue was replaced with tartrazine (λ_max_ = 435 nm), a water and ethanol-soluble dye. A new batch of implants was prepared with tartrazine and the release characteristics investigated in vitro (Table 2 and Supplementary Materials Figure S3). Implants containing a 1080 core formulation and tartrazine exhibited similar release characteristics and release profiles to the NaOAc implants (Supplementary Materials Table S2), which confirmed that metachromasy between methylene blue and 1080 was the cause of the protracted release profiles. However, a greater build-up of electrostatic charge was observed during the manufacture of the tartrazine core implants, leading to end-defects which precluded their further investigation (Supplementary Materials Figure S4). 

### 3.4. In Vitro Stability

**Figure 5:**
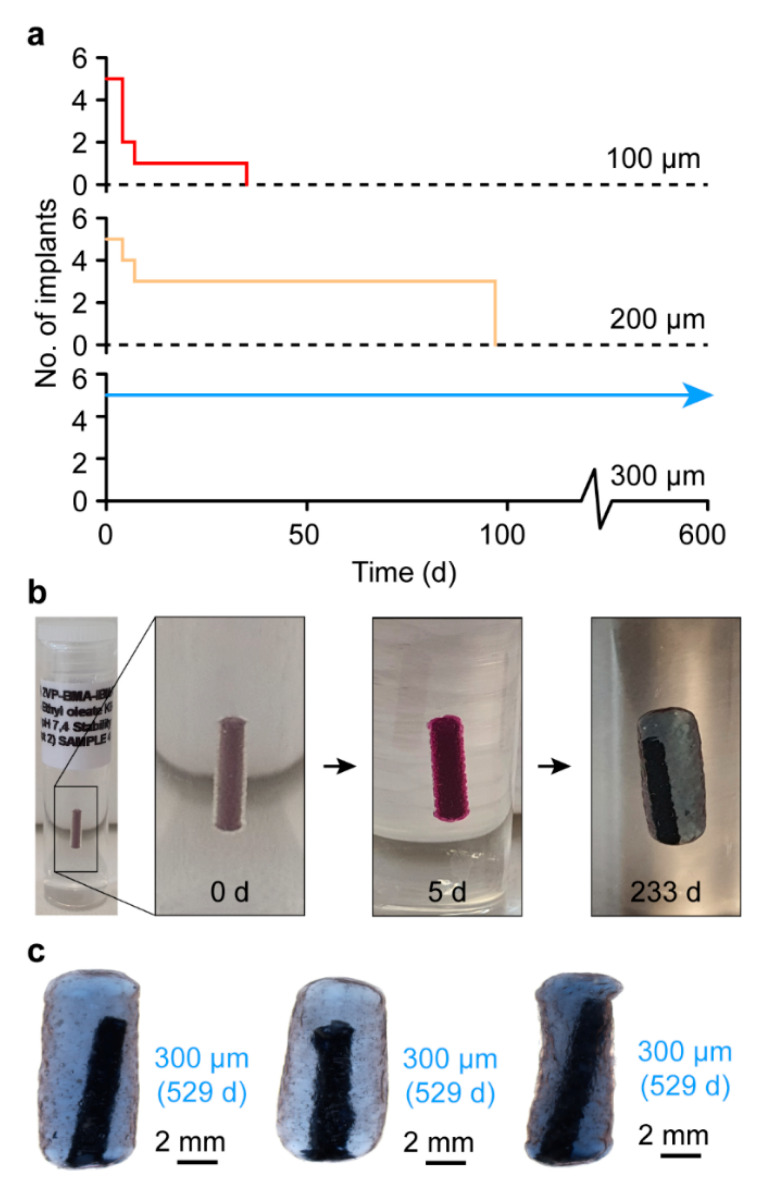
(**a**) Stability results of 1080 core and PVBI/EO_10_-coated implants (100, 200, or 300 µm) in Pluronics F127 (25 wt%, in 10 mM PBS) at pH 7.4 and 37 °C (*n* = 6), over 529 d. Images showing (**b**) the swelling of a 300 µm 1080 core and PVBI/EO_10_-coated implant over 233 d, and (**c**) representative implants that were stable at 529 d.

The stability of the 1080 core implants in a simulated subcutaneous environment was investigated in vitro by embedding the implants in a hydrogel tissue mimic [20]. 1080 implants bearing a 100 or 200 µm PVBI/EO_10_ coating displayed poor stability, with all the implants failing by 35 and 97 d in vitro, respectively (Figure 5a). In contrast, 1080 implants with a 300 µm PVBI/EO_10_ coating were stable for the duration of the study (529 d), similar to the NaOAc implants with the same coating thickness. The same swelling behaviour was observed for the 1080 implants (*cf.* NaOAc implants) (Figure 5b,c), which was anticipated given the similar physicochemical properties of 1080 and NaOAc and the use of the same semi-permeable PVBI/EO_10_ coating. However, it is likely that the failures observed for the 100 and 200 µm implants were due to the flared ends, as seen during the optical microscopy and SEM imaging of whole and microtomed implants (Figure 1). While the coatings appeared uniform in thickness, the asymmetric shapes of the implant ends appeared to undergo a non-uniform swelling, which may have resulted in greater local mechanical stresses that caused the polymer coating to rupture and the implants to fail.

At thicker PVBI/EO_10_ coatings, the implants exhibited a more uniform morphology, which likely afforded more uniform swelling and a reduced chance of failure, as evidenced by the proportional relationship between coating thickness and implant stability. It was unclear what caused the flaring of the implant ends, but further investigation is warranted considering that flaring was also observed when coating the 1080 core implants containing tartrazine. The observed flaring for the 1080 core implants containing either methylene blue or tartrazine indicated that this phenomenon was related to the physicochemical properties of 1080. Regardless, similar to their NaOAc counterparts, 1080 core implants possessing a 300 µm PVBI/EO_10_ coating exhibited promising stability and were considered suitable for further testing. 

### 3.5. Assessment of 1080 Diffusion In Vitro

The in vitro stability of the 1080 core implants was determined via the observed containment of methylene blue within the core during testing, which served as a proxy for all core materials. It was considered that at physiological pH, it was highly unlikely that the anionic 1080, or comparatively high molecular weight PEG 10 kDa, would diffuse across the semi-permeable PVBI/EO_10_ coating. However, the effective containment of 1080 within the implant core in vivo was crucial to ensure a lethal dose of 1080 would be present if the implant was consumed by a feral cat. In addition, the diffusion of 1080 from an implant in vivo could result in the toxification of the native mammal bearing it—though only if the species is particularly susceptible to 1080—and should be avoided. As a result, an accelerated in vitro stability study was conducted to determine whether 1080 would diffuse following swelling.

1080 implants were submerged in PBS (pH 7.4) and stored at 37 °C for 296 d. The receiving solutions were analysed periodically via HPLC for the presence of 1080 (λ = 220 nm) and quantified, where detected (Supplementary Materials Figure S5). 1080 implants at each coating thickness (100, 200, and 300 µm) (*n* = 3) were tested. However, all the 100 µm, and two 200 µm PVBI/EO_10_ coated 1080 implants failed within 4 d, as indicated by the release of methylene blue into the receiving solution (Supplementary Materials Figure S6). The failures occurred before the implants had swelled significantly, which precluded their analysis. The remaining 200 and all the 300 implants appeared intact for the duration of the experiment, with no observed release of methylene blue (Supplementary Materials Figure S7). A single 300 µm PVBI/EO_10_ coated implant was found to release 1080 into the receiving solution. Cumulatively, 3.08 mg of 1080 was released into the receiving solution, which was equivalent to 98.7% of the total theoretical loading (Supplementary Materials Figure S8). In contrast, no 1080 was detected from the remaining implants. If the diffusion of 1080 through the PVBI/EO_10_ coating had occurred, it was expected that it would be detected in all samples once in a swollen state, independent of coating thickness. However, since 1080 was only detected for a single sample, and at every time point, it was likely a result of the failure of the PVBI/EO_10_ coating shortly after swelling, and not diffusion. These results demonstrated that for both in vitro and in vivo stability experiments, 1080 was effectively contained within the intact PVBI/EO_10_ coatings of the 1080 implants. 

### 3.6. In Vivo Stability

**Figure 6:**
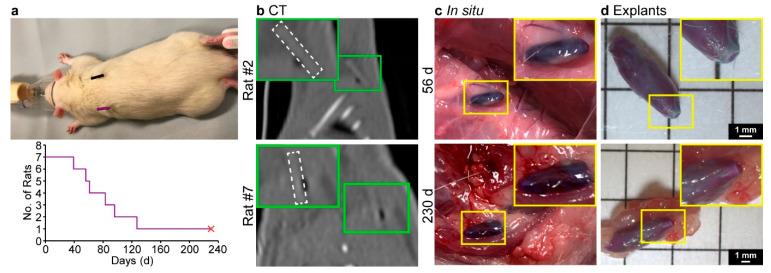
(**a**) Implant (purple) and microchip (black) locations (top), and number of surviving rats over time (bottom); the body masses of the rats were monitored throughout the study (Supplementary Materials Figure S9). (**b**) CT images captured immediately following implantation (i.e., day 0), with implants outlined in white. (**c**) Representative macroscopic images (post-mortem) of implants in situ captured prior to explantation, after 56 d (failed) (top) and 230 d (stable) (bottom). (**d**) Representative optical microscopy images of explants after 56 (failed) (top) and 230 d (stable) (bottom) taken on a 5 × 5 mm grid; note the protrusion of the ABS filament scaffold through the polymer coating in both samples. Additional images of the remaining explants are available in the SI (Figures S10 and S11).

The in vivo stability of the 1080 core implants with a 300 µm PVBI/EO_10_ coating was assessed following subcutaneous injection in rats over 230 d [20]. Male Sprague-Dawley rats (*n* = 7) were each implanted (in reverse time order) with one 1080 core implant, and one RFID microchip (control), superficial to the left and right scapula, respectively (Figure 6a, top). Body weight measurements were recorded weekly to monitor rat wellbeing (Supplementary Materials Figure S9), and implant integrity was qualitatively monitored via computed tomography (CT) immediately following implantation (Figure 6b and Supplementary Materials Figure S10a) and every 6 weeks thereafter (Supplementary Materials Figure S11). CT images recorded of the implants (12- and 18-weeks post-implantation) revealed that considerable swelling had occurred for all implants following implantation (Supplementary Materials Figure S11). Following implantation, at 39 d Rat #1 was found deceased. Post-mortem examination of the 1080 implant revealed it had failed in situ with the core material observed leaking from the fibrous tissue capsule that had formed. Similarly, 56 and 61 d after implantation Rats #2 and #3 had lost body mass and were exhibiting signs of 1080 poisoning (e.g., ataxia, tremors, hunched posture, and hypothermia), and were euthanised. 1080 toxicosis was confirmed through analysis of liver tissues from Rats #1 and #2, where 1080 was detected in concentrations of 2.0 and 0.87 mg·kg^−1^, respectively. This trend continued, with two more deaths after 83 and 96 d (Rats #4 and #5), and another rat (#6) euthanised 127 d following implantation due to 1080 toxicosis (Figure 6a, bottom). The surviving rat (#7) exhibited no signs of 1080 toxicosis and was euthanised 230 d after implantation to conclude the study. 

These results clearly demonstrated that the 1080 core implants exhibited poor stability in vivo, despite the favourable in vitro stability results, and in contrast to the favourable in vitro/in vivo stability of the NaOAc core implants [20]. No marked inflammation was observed during post-mortem examination/explanation at any time point, and a fibrous tissue capsule formed around all implants (Figure 6c and Figure S10). The NaOAc core implants exhibited increased fibrous capsule thicknesses from week 1 to 2 and were constant thereafter, commensurate with the expected changes in cell populations (i.e., the formation and maturation of granulation tissue into a fibrous capsule) [20,55]. These changes indicated that the NaOAc core implants exhibited comparable biocompatibility to RFID microchips, which served as a control. Considering the 1080 core implants bear the same PVBI/EO_10_ coating and the tissue reaction was indistinguishable at a gross level, the 1080 implants core implants appeared to also exhibit favourable biocompatibility. Importantly, histological examination of the implants was not conducted, as suitable biocompatibility had already been established. Instead, post-mortem examinations of the implants (Figure 6c) and optical microscopy of the explants (Figure 6d) were undertaken to interrogate the mechanism of 1080 core implant failure. While the implants appeared intact in situ, inspection of the explants showed that the ABS filament scaffold had pierced the polymer coating following swelling, causing the premature release of 1080 and the toxification of the rats. It is likely that once in a swollen state, the ABS filament was able to float freely within the solubilised 1080 core and pierced the polymer coating when movement of the implanted animal caused sufficient pressure at the implant location. However, this occurred inconsistently as the final implant remained intact, with the 1080 effectively contained, at the conclusion of the experiment (230 d). Interestingly, when removed from the fibrous tissue capsule and broken, the PVBI/EO_10_ coatings of the implants were discoloured and appeared similar in colour to the solutions of the in vitro release experiments (Supplementary Materials Figure S10c,d). It appeared that in the presence of 1080, methylene blue (likely in an aggregated state) had interacted with and stained the PVBI/EO_10_ coating. The extent of this interaction and the mechanism through which it occurred was not immediately apparent. However, it was hypothesised that it could have led to a change in the physical properties of the coating, possibly making it more brittle and prone to fracture, as this effect was not observed in the NaOAc implants [20].

It was previously thought that implant location contributed to the failure of some NaOAc implants in vivo [20]. However, the data reported herein suggested that the failures were likely primarily due to the ABS filament scaffold and its rupturing of the PVBI/EO_10_ coating. In addition, the integrity of the PVBI/EO_10_ coating may have been reduced by the aggregates of methylene blue in the swollen 1080 core and contributed to the observed failures.

## 4. Conclusions

Population Protecting Implants with poison-loaded cores (containing a lethal dose of 1080 for feral cats, ≈3.12 mg) and pH-responsive polymer coatings were manufactured via fluidised-bed spray coating with low intra-batch variability. The implants had cohesive and uniform coatings as demonstrated by optical microscopy and scanning electron microscopy. 

The in vitro stability of the 1080 core implants bearing a 300 µm polymer coating was found to be favourable in a hydrogel model of the subcutaneous environment, despite water permeation and swelling of the core. Diffusion of 1080 through the polymer coating of the swollen implants was not detected and confirmed that 1080 was contained effectively within the swollen core. However, in vitro release experiments conducted under simulated gastric conditions, were complicated by the metachromasy exhibited by methylene blue. Aggregation of methylene blue cations during release from the core, was likely caused by the presence of—and possible interactions with—1080. Regardless, release experiments conducted on implants containing the non-metachromatic dye tartrazine in the core, demonstrated that the complete release of 1080 from the core was likely to occur within the gastric emptying time of a feral cat. 

The in vivo stability of the 1080 core implants was limited, primarily due to the presence of the filament scaffold in the core, and possibly affected by a reduction in mechanical integrity of the polymer coating, caused by an interaction between the coating and methylene blue aggregates. Previous in vivo experiments involving NaOAc core implants exhibited greater stability, likely due to comparatively minor interactions between NaOAc and methylene blue, and reduced effects on the integrity of the polymer coating. 

The filament scaffold was found to have punctured the polymer coatings of almost all the tested implants, causing inconsistent implant stability and rat survival, which ranged from ~ 39–230 d. To improve the performance of future implants, a water-soluble implant scaffold containing a non-metachromatic dye could be used. Replacing methylene blue with a non-metachromatic dye would eliminate any potential reduction in mechanical integrity of the coating caused by metachromasy. However, removal of the ABS filament scaffold is also necessary to prevent the puncturing of the polymer coating in vivo. A water-soluble core would solubilise during swelling, which the PVBI/EO_10_ coating appears able to accommodate. The coating may then remain intact and stable for a sufficient period and reduce the likelihood of failure in vivo.

## Figures and Tables

**Table 1:** Summary of implant dimensions at various stages during manufacture.

Coating Thickness (µm)	Diameter (mm) [$\bar{x}$ ± σ] ^a^	Length (mm) [$\bar{x}$ ± σ] ^a^	Mass (mg) [$\bar{x}$ ± σ] ^a^	Coating Thickness (Calliper) (µm) [$\bar{x}$ ± σ] ^a^		Coating Thickness (Microtomed) (um) [$\bar{x}$ ± σ] ^b^			
				Side Midpoint	End	Overall ^c^	Ends ^d^	Sides ^e^	Side Midpoint ^f^
1080 Core	1.04 ± 0.05 (4.8)	7.79 ± 0.09 (1.2)	9.30 ± 0.74 (8.0)	204 ± 8 (3.9)	200 ± 12 (6.0)	235 ± 31 (13.2)	238 ± 41 (17.2)	234 ± 20 (8.5)	235 ± 16 (6.8)
100	1.29 ± 0.04 (3.1)	8.07 ± 0.08 (1.0)	12.08 ± 0.74 (6.1)	125 ± 7 (5.6)	141 ± 15 (9.9)	132 ± 13 (9.8)	138 ± 13 (9.8)	127 ± 9 (7.1)	129 ± 6 (4.7)
200	1.43 ± 0.03 (2.1)	8.15 ± 0.11 (1.3)	14.76 ± 0.68 (4.6)	195 ± 7 (3.6)	181 ± 18 (9.9)	186 ± 12 (6.5)	186 ± 14 (7.5)	185 ± 10 (5.4)	183 ± 11 (6.0)
300	1.63 ± 0.04 (2.5)	8.34 ± 0.11 (1.3)	19.41 ± 0.79 (4.1)	295 ± 7 (2.4)	276 ± 18 (6.5)	246 ± 15 (6.1)	245 ± 18 (7.3)	247 ± 13 (5.3)	241 ± 12 (5.0)

All of the results are reported as the mean ± std. dev.; %RSD values are reported in parentheses. ^a^ Determined from *n* ≥ 50 digital calliper measurements; ^b^ Determined from *n* = 3 (1080 core, 100, and 200 µm) and *n* = 6 (300 µm) epoxy-embedded and microtomed implants; ^c^ Determined from 22 point-to-point measurements; ^d^ Determined from 10 point-to-point measurements; ^e^ Determined from 12 point-to-point measurements; ^f^ Determined from 2 point-to-point measurements.

**Table 2:** Summary of 1080-implant in vitro release results when the core contained methylene blue or tartrazine.

Coating Thickness (µm) ^a^	pH 1.0				pH 1.5			
	Initial Release (min) ^b^		Complete Release (min) ^c^		Initial Release (min) ^b^		Complete Release (min) ^c^	
	Methylene Blue	Tartrazine	Methylene Blue	Tartrazine	Methylene Blue	Tartrazine	Methylene Blue	Tartrazine
100	7.0 ± 0.7	8.2 ± 0.4	37.8 ± 12.5	41.9 ± 5.4	15.5 ± 2.2	21.6 ± 0.9	155.0 ± 22.0	38.5 ± 2.5
200	9.6 ± 0.5	7.9 ± 0.6	24.6 ± 10.6	32.6 ± 5.5	26.4 ± 1.6	37.1 ± 6.0	184.8 ± 18.0	65.7 ± 3.6
300	15.0 ± 0.5	11.3 ± 0.3	37.4 ± 9.8	33.1 ± 2.1	52.2 ± 2.6	45.4 ± 5.4	213.8 ± 18.1	118.4 ± 9.2

All values are reported as the mean ± std. dev. (*n* = 5). ^a^ 1080-implants coated with the PVBI/EO_10_ formulation; ^b^ Initial release was defined as 5% of the total release of dye (methylene blue or tartrazine) from the implant core; ^c^ Complete release was defined as 90% of the total release of dye (methylene blue or tartrazine) from the implant core.

## Data Availability

Raw data supporting the findings of this study are available from the corresponding author, upon reasonable request.

## Supplementary Materials

The following supporting information can be downloaded at: https://www.mdpi.com/article/10.3390/polym15040878/s1, Table S1: Summary of two-tailed Student’s *t*-test results; Figure S1: Representative SEM micrographs of 1080 core-coated and NaOAc core-coated implants; Equation S1: Determination of implant flexural strength; Equation S2: Determination of implant flexural modulus; Figure S2: Force versus stroke graphs obtained via three-point flexural testing of 1080 core and PVBI/EO_10_-coated implants; Table S2: Summary of NaOAc core and PVBI/EO_10_-coated implant in vitro release results; Figure S3: Release monitoring profiles of tartrazine from PVBI/EO_10_-coated 1080/tartrazine core implants; Figure S4: Representative digital images and SEM micrographs of tartrazine/1080 core and PVBI/EO_10_-coated implants; Figure S5: Representative HPLC chromatograms of 1080 standard solutions and 1080 diffusion samples; Figure S6: Digital images of PVBI/EO_10_-coated 1080 core implants that failed under accelerated swelling conditions; Figure S7: Representative digital images of PVBI/EO_10_-coated 1080 core implants captured under accelerated swelling conditions; Figure S8: Cumulative release profile of 1080 from a failed PVBI/EO_10_-coated 1080 core implant under accelerated swelling conditions; Figure S9: Rat body masses recorded during the in vivo stability experiment of PVBI/EO_10_-coated 1080 core implants; Figure S10: Summary of images captured during the in vivo stability study of PVBI/EO_10_-coated 1080 core implants; Figure S11: CT scans of PVBI/EO_10_-coated 1080 core implants.
