# Supplementary material for: pH-Responsive Polymer Implants for the Protection of Native Mammals: Assessment of Material Properties and Poison Incorporation on Performance
Source: Polymers (Basel). 2023 Feb 10;15(4):878. doi: 10.3390/polym15040878 (PMC9958913; doi:10.3390/polym15040878)
Supplement: Supplementary file 1 [file polymers-15-00878-s001.zip › polymers-2154543-supplementary.pdf]

polymers

MDPI

Supplementary Materials

pH-Responsive Polymer Implants for the Protection of Native Mammals: Assessment of Material Properties and Poison Incorporation on Performance

Kyle Brewer [1], Todd J. McWhorter [2], Katherine Moseby [3,4], John L. Read [3,5], David Peacock [6] and Anton Blencowe [1,*]

[1] Applied Chemistry and Translational Biomaterials (ACTB) Group, Centre for Pharmaceutical Innovation (CPI), UniSA Clinical and Health Sciences, University of South Australia, Adelaide, SA 5000, Australia; kyle.brewer@mymail.unisa.edu.au
[2] School of Animal and Veterinary Sciences, University of Adelaide, Roseworthy, SA 5371, Australia; todd.mcwhorter@adelaide.edu.au
[3] Ecological Horizons Pty. Ltd., P.O. Box 207, Kimba, SA 5641, Australia; katherine.moseby@adelaide.edu.au (K.M.); ecological67@gmail.com (J.L.R.)
[4] School of Biological, Earth and Environmental Sciences, University of New South Wales, Kensington, Sydney, NSW 2033, Australia
[5] School of Biological Sciences, University of Adelaide, Adelaide, SA 5000, Australia
[6] Davies Livestock Research Centre, School of Animal and Veterinary Sciences, University of Adelaide, Roseworthy, SA 5371, Australia; david.peacock@adelaide.edu.au
* Correspondence: anton.blencowe@unisa.edu.au

Citation: Brewer, K.; McWhorter, T.J.; Moseby, K.; Read, J.L.; Peacock, D.; Blencowe, A. pH-responsive Polymer Implants for the Protection of Native Mammals: Assessment of Material Properties and Poison Incorporation on Performance. *Polymers* **2023**, *15*, 878. https://doi.org/10.3390/polym15040878

Academic Editors: Mirosława El Fray and Deng-Guang Yu

Received: 27 December 2022
Revised: 2 February 2023
Accepted: 6 February 2023
Published: 10 February 2023

Copyright: © 2023 by the authors. Licensee MDPI, Basel, Switzerland. This article is an open access article distributed under the terms and conditions of the Creative Commons Attribution (CC BY) license (https://creativecommons.org/licenses/by/4.0/).

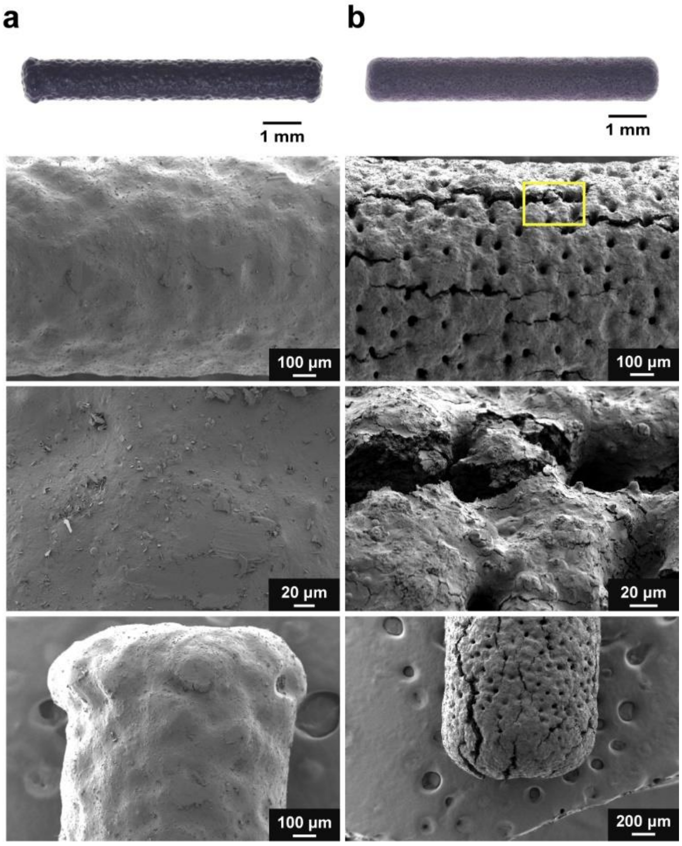

Figure S1. Representative scanning electron micrographs of a) 1080 core-coated and b) NaOAc core-coated implants. Note the reduced porosity and cracking observed in the 1080 core implants, and the presence of flared ends.

Table S1. Summary of two-tailed Student's t-test results (t-test of difference = 0 (vs. ≠).

Test of Mean	T-value	Degrees of freedom (DF)	P-value
Flexural strength of PVBI/EO_{10}-coated NaOAc- vs. 1080-core implants (100 μm)	-0.03	7	0.973
Flexural strength of PVBI/EO_{10}-coated NaOAc- vs. 1080-core implants (200 μm)	1.26	5	0.265
Flexural strength of PVBI/EO_{10}-coated NaOAc- vs. 1080-core implants (300 μm)	0.02	4	0.988
Flexural modulus of PVBI/EO_{10}-coated NaOAc- vs. 1080-core implants (100 μm)	0.44	6	0.679
Flexural modulus of PVBI/EO_{10}-coated NaOAc- vs. 1080-core implants (200 μm)	2.62	7	0.035
Flexural modulus of PVBI/EO_{10}-coated NaOAc- vs. 1080-core implants (300 μm)	0.34	7	0.743

Equation S1: Determination of implant flexural strength (σ_F).

$$\sigma_F = \frac{8F_{max}l}{\pi d^3}$$

where *d* (mm) is the diameter of the implant, F_{max} (N) is the maximum force exerted on the implant prior to failure, *l* (mm) is the support span of the flexural testing apparatus.

Equation S2: Determination of implant flexural modulus (E_f).

$$E_f = \frac{4F_{max}l^3}{D3\pi d^4}$$

where *D* (mm) is the deflection at F_{max} determined from the linear region of the force versus stroke curve.

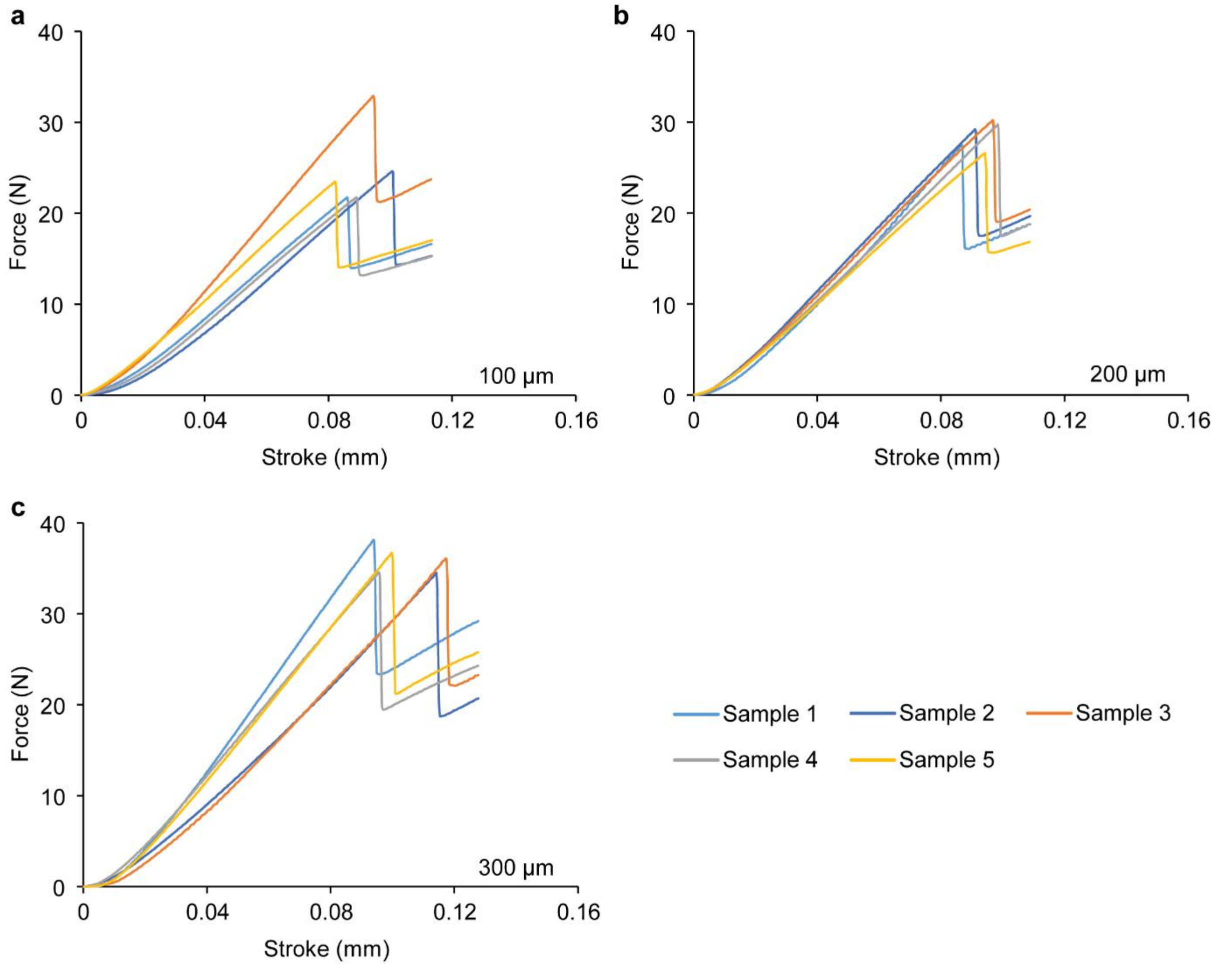

Figure S2. Force versus stroke graphs obtained *via* three-point flexural testing of 1080 core implants with a) 100, b) 200 and c) 300 μm PVBI/EO_{10} coatings (n = 5 for all samples).

Table S2. Summary of NaOAc core and PVBI/EO_{10} coated implant *in vitro* release results.[1].

Coating Thickness (μm) [a]	pH 1.0		pH 1.5	
	**Initial Release (min) [b]**	**Complete Release (min) [c]**	**Initial Release (min)**	**Complete Release (min)**
**100**	10.0 ± 0.5	15.9 ± 0.6	34.0 ± 1.4	54.2 ± 4.1
**200**	12.21 ± 0.9	19.6 ± 1.9	39.9 ± 1.3	77.4 ± 6.7
**300**	16.6 ± 1.2	26.8 ± 1.6	53.0 ± 2.6	99.3 ± 6.7

All values are reported as the mean ± std. dev. (n = 5). [a] NaOAc core implants coated with the PVBI/EO_{10} formulation; [b] Initial release was defined as 5% of the total release of methylene blue ($\lambda_{max.}$ = 664 nm) from the implant core; [c] Complete release was defined as 90% of the total release of methylene blue from the implant core.

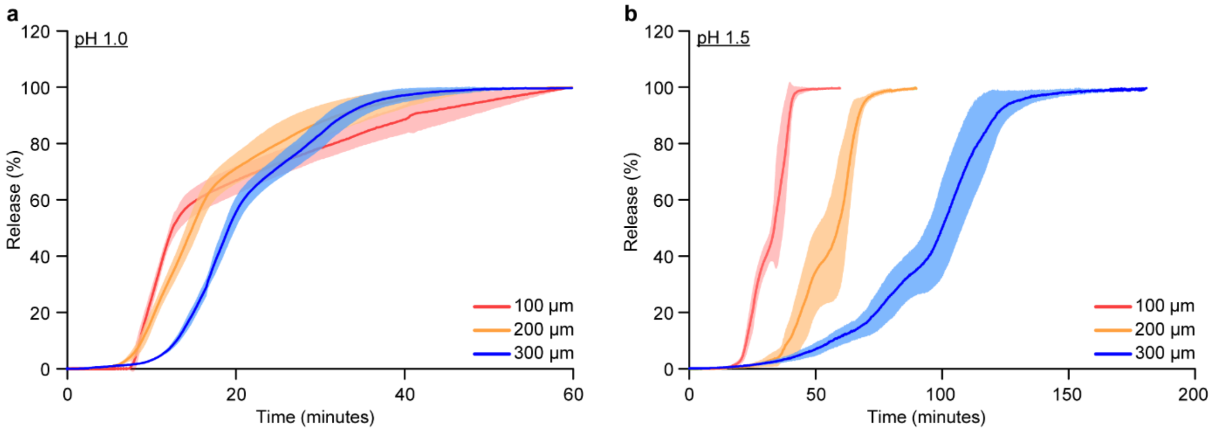

Figure S3. Release monitoring profiles of tartrazine from PVBI/EO_{10}-coated 1080/tartrazine core implants (100, 200 or 300 µm) at 37 ± 1 °C, in pH 1.0 or pH 1.5 (3.5 mL) release media. Traces are reported as the mean (solid line) ± std. dev. (shaded area) (n = 5).

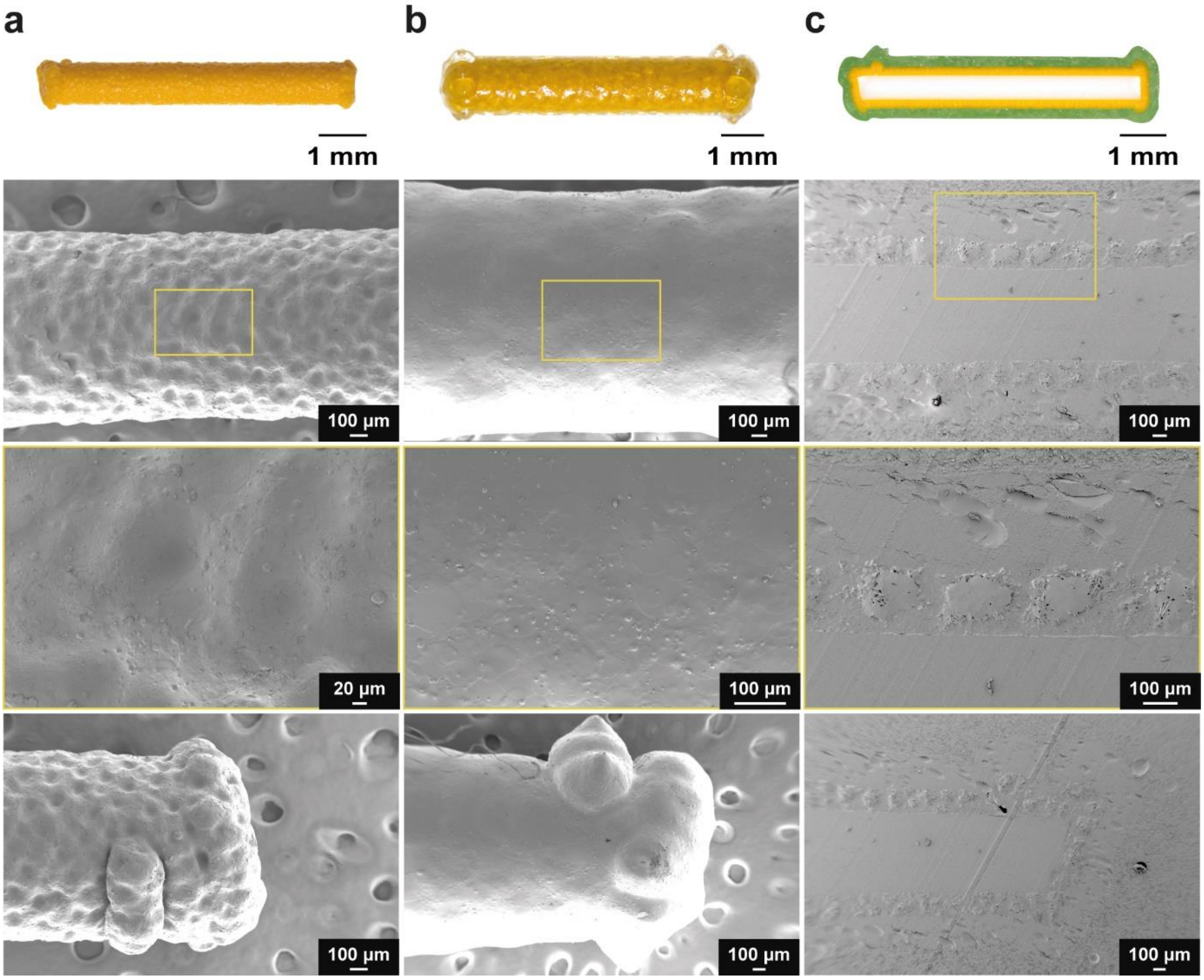

Figure S4. Representative digital images and scanning electron micrographs of a) a tartrazine/1080 core-coated implant, b) a tartrazine/1080 core and PVBI/EO_{10}-coated (300 µm) implant, and c) an epoxy-embedded and microtomed tartrazine/1080 core and PVBI/EO_{10}-coated (300 µm) implant.

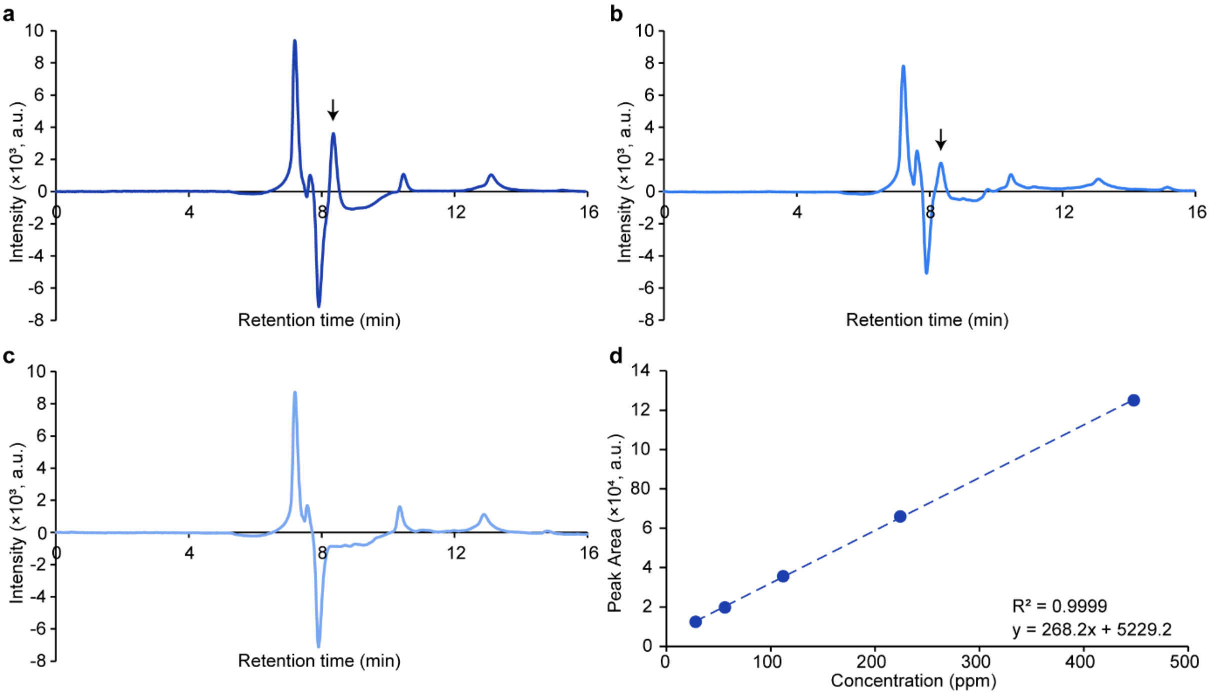

Figure S5. Representative high-performance liquid chromatography chromatograms of a) a 224 ppm 1080 standard solution prepared in PBS (pH 7.4) used in the generation of a standard curve, b) the presence of 1080 and c) the absence of 1080, determined in the receiving solution (PBS, pH 7.4) of respective PVBI/EO_{10}-coated (300 µm) implants at 173 d. The peaks attributed to 1080 are indicated where present with arrows.

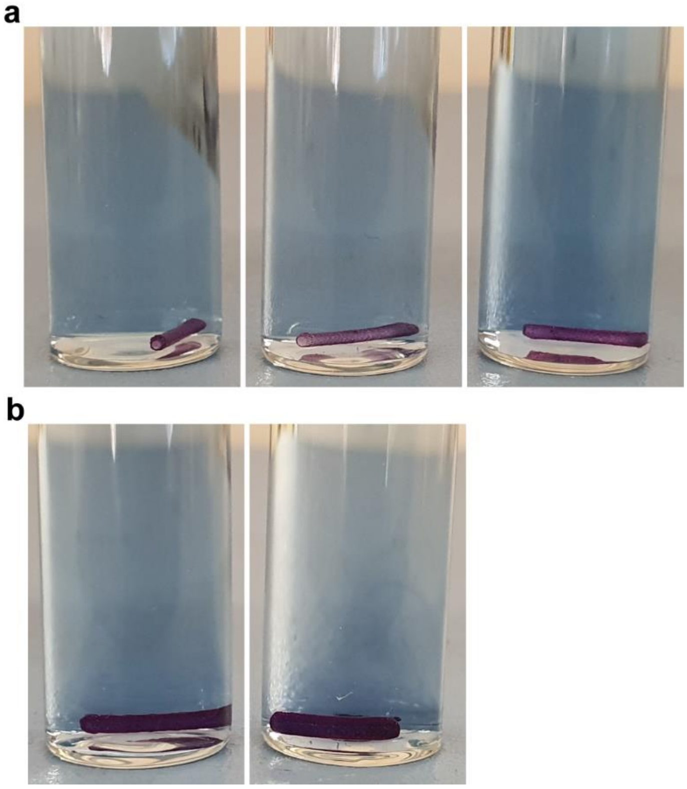

Figure S6. Digital images of the 1080 core and a) 100 µm and b) 200 µm PVBI/EO_{10}-coated implants that failed under accelerated swelling conditions (PBS, PH 7.4, 37 °C).

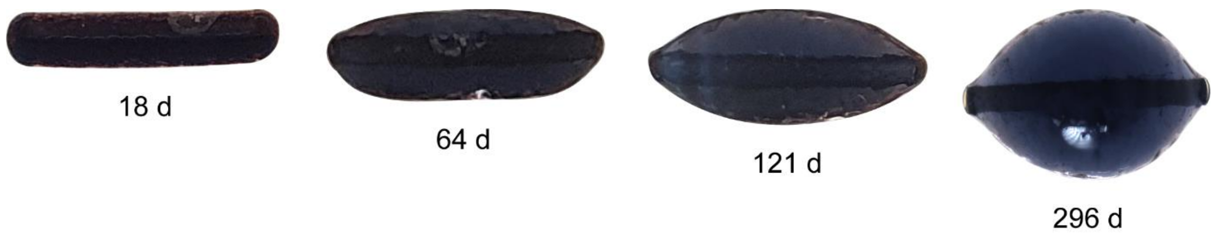

Figure S7. Representative digital images of a 1080 core and PVBI/EO10-coated (300 μm) implant captured under accelerated swelling conditions (PBS, pH 7.4, 37 °C).

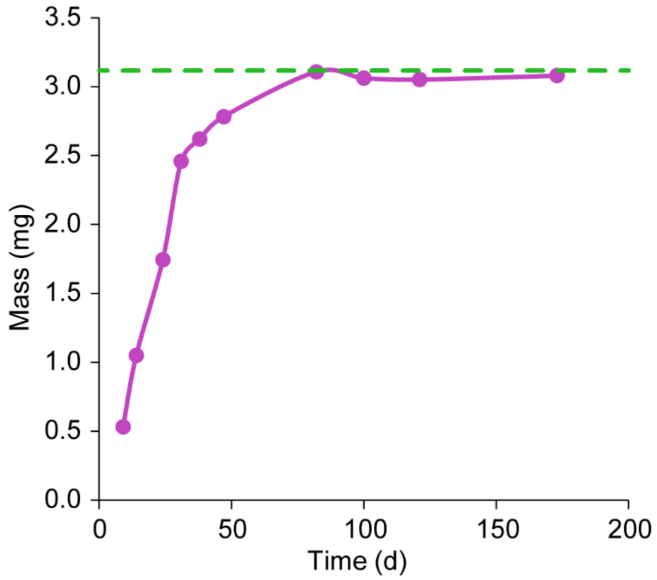

Figure S8. Cumulative release profile of 1080 from a failed PVBI/EO10-coated (300 μm) implant under accelerated swelling conditions (PBS, pH 7.4, 37 °C). The presence of 1080 was determined *via* HPLC (λ = 220 nm). Note the dashed line indicates the average calculated loading (3.12 mg) of 1080.

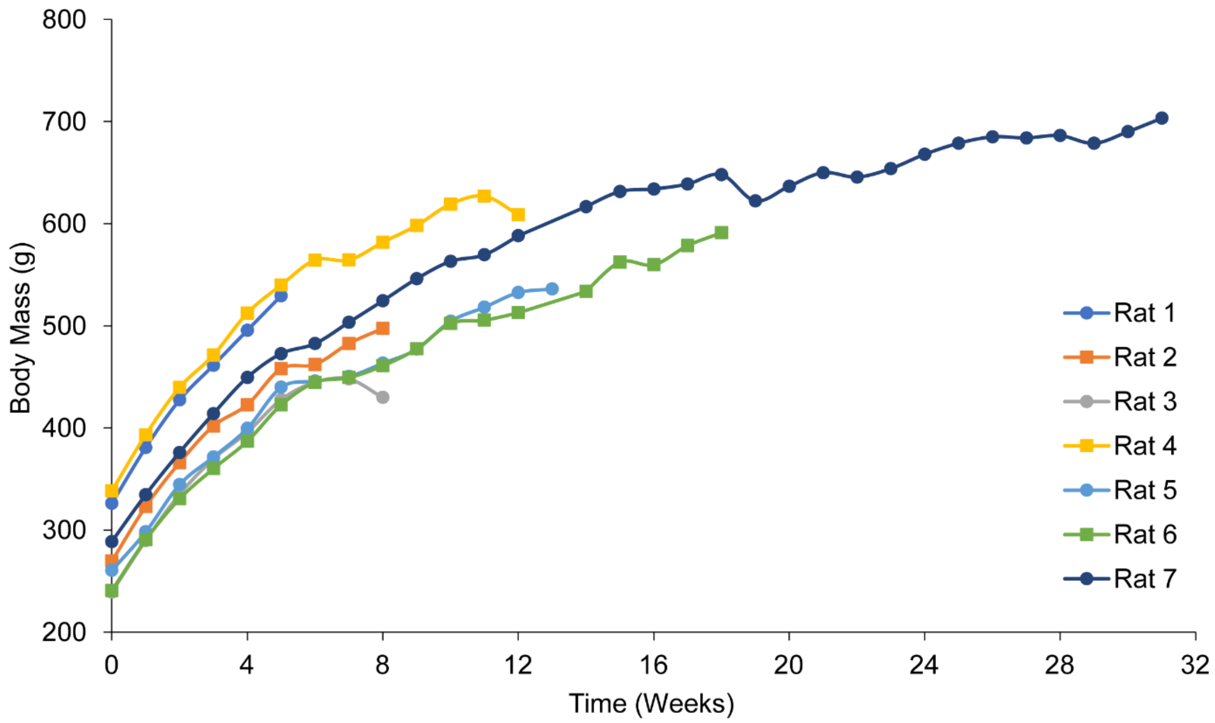

Figure S9. Rat body masses (n = 7), recorded during the *in vivo* stability experiment of the 1080 core and PVBI/EO10-coated (300 μm) implants.

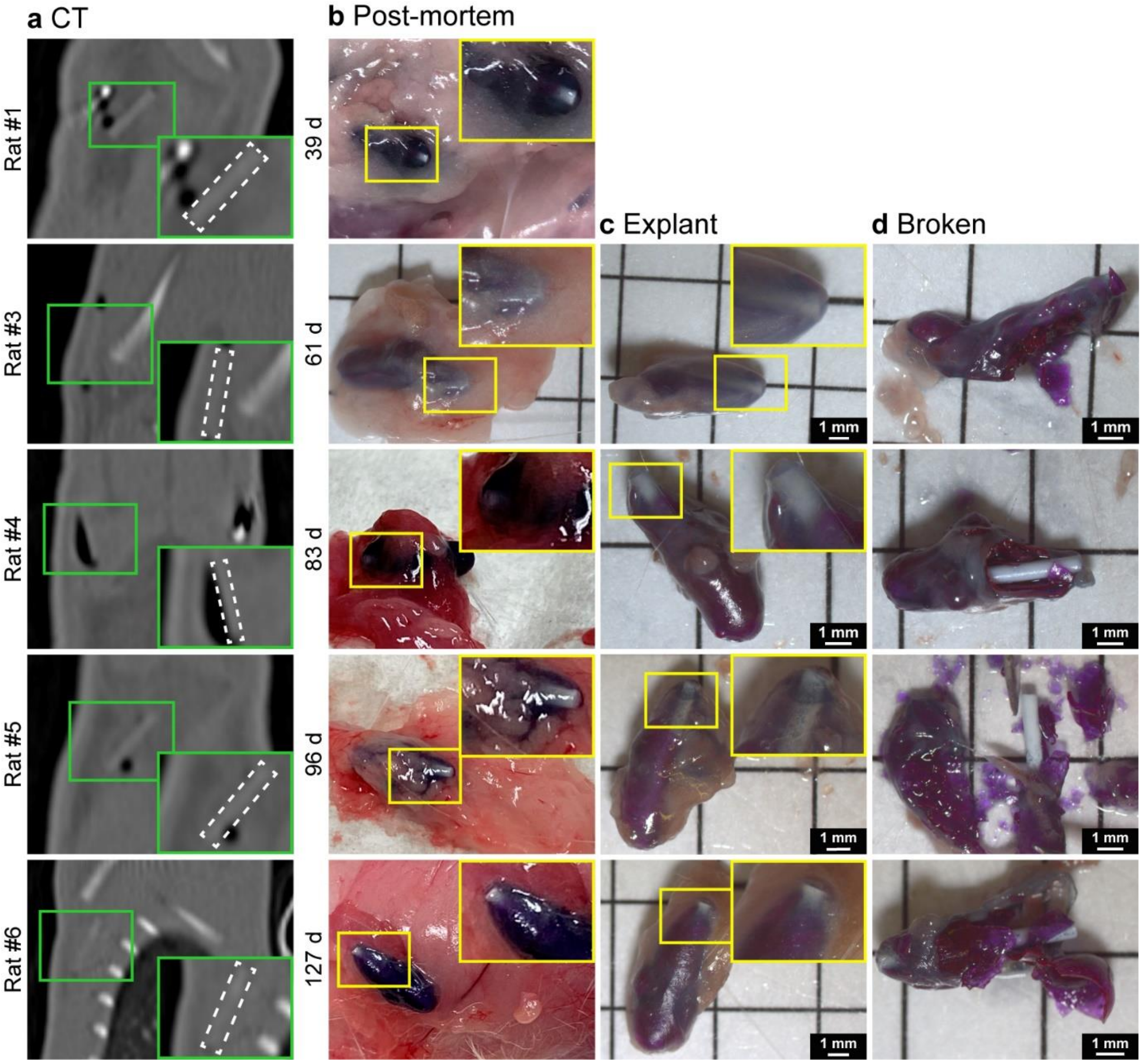

Figure S10. A summary of images captured during the *in vivo* stability study of the 1080 core implants bearing a PVBI/EO_{10} (300 μm) coating. a) CT images captured immediately following implantation (i.e., day 0), note the implants outlined in white. Photographs captured b) during post-mortem examination *in* or *ex situ*, c) following explantation and the removal of most of the surrounding subcutaneous tissues, and d) following dissection of the fibrous tissue capsule and breakage of the explant. Note, the 5 × 5 mm grid used in the images of b) and c).

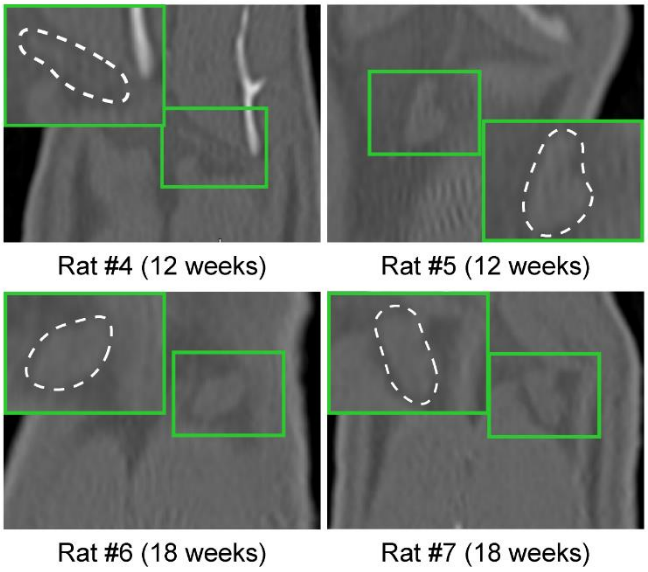

Figure S11. CT scans of 1080 core and PVBI/EO_{10}-coated (300 μm) implants recorded at the latest time point available prior to failure *in vivo*, with implants outlined in white.

References

1. Brewer, K.; McWhorter, T.J.; Sloper, C.; Moseby, K.; Read, J.L.; Peacock, D.; Blencowe, A. Toward Targeted Invasive Predator Control: Developing pH-Responsive Subcutaneous Implants for Native Mammals. *ACS Appl. Polym. Mater.* **2022**, *4*, 6687–6699, https://doi.org/10.1021/acsapm.2c01041.

Disclaimer/Publisher's Note: The statements, opinions and data contained in all publications are solely those of the individual author(s) and contributor(s) and not of MDPI and/or the editor(s). MDPI and/or the editor(s) disclaim responsibility for any injury to people or property resulting from any ideas, methods, instructions or products referred to in the content.
